# Supplementary material for: A fault-intrusion-tolerant system and deadline-aware algorithm for scheduling scientific workflow in the cloud
Source: PeerJ Comput Sci. 2021 Nov 2;7:e747. doi: 10.7717/peerj-cs.747 (PMC8576554; doi:10.7717/peerj-cs.747)
Supplement: Supplemental Information 6 [file peerj-cs-07-747-s006.zip › Myproject1.1/docs/org/cloudbus/cloudsim/examples/power/Helper.html]

Helper


JavaScript is disabled on your browser.


- Overview
- Package
- Class
- Use
- Tree
- Deprecated
- Index
- Help

- Prev Class
- Next Class

- Frames
- No Frames

- All Classes

- Summary:
- Nested |
- Field |
- Constr |
- Method

- Detail:
- Field |
- Constr |
- Method


org.cloudbus.cloudsim.examples.power

## Class Helper

- java.lang.Object
- - org.cloudbus.cloudsim.examples.power.Helper

- ---

    

  ```
  public class Helper
  extends java.lang.Object
  ```

  The Class Helper.
  If you are using any algorithms, policies or workload included in the power package, please cite
  the following paper:
  Anton Beloglazov, and Rajkumar Buyya, "Optimal Online Deterministic Algorithms and Adaptive
  Heuristics for Energy and Performance Efficient Dynamic Consolidation of Virtual Machines in
  Cloud Data Centers", Concurrency and Computation: Practice and Experience (CCPE), Volume 24,
  Issue 13, Pages: 1397-1420, John Wiley & Sons, Ltd, New York, USA, 2012

- - ### Constructor Summary

    Constructors

    Constructor and Description

    `Helper()`
  - ### Method Summary

    Methods

    | Modifier and Type | Method and Description |
    | --- | --- |
    | `static DatacenterBroker` | `createBroker()` Creates the broker. |
    | `static Datacenter` | `createDatacenter(java.lang.String name, java.lang.Class<? extends Datacenter> datacenterClass, java.util.List<PowerHost> hostList, VmAllocationPolicy vmAllocationPolicy)` Creates the datacenter. |
    | `static java.util.List<PowerHost>` | `createHostList(int hostsNumber)` Creates the host list. |
    | `static java.util.List<Vm>` | `createVmList(int brokerId, int vmsNumber)` Creates the vm list. |
    | `protected static java.util.Map<java.lang.String,java.lang.Double>` | `getSlaMetrics(java.util.List<Vm> vms)` Gets the sla metrics. |
    | `protected static double` | `getSlaTimePerActiveHost(java.util.List<Host> hosts)` Gets the sla time per active host. |
    | `protected static double` | `getSlaTimePerHost(java.util.List<Host> hosts)` Gets the sla time per host. |
    | `static java.util.List<java.lang.Double>` | `getTimesBeforeHostShutdown(java.util.List<Host> hosts)` Gets the times before host shutdown. |
    | `static java.util.List<java.lang.Double>` | `getTimesBeforeVmMigration(java.util.List<Vm> vms)` Gets the times before vm migration. |
    | `static java.lang.String` | `parseExperimentName(java.lang.String name)` Parses the experiment name. |
    | `static void` | `printCloudletList(java.util.List<Cloudlet> list)` Prints the Cloudlet objects. |
    | `static void` | `printMetricHistory(java.util.List<? extends Host> hosts, PowerVmAllocationPolicyMigrationAbstract vmAllocationPolicy)` Prints the metric history. |
    | `static void` | `printResults(PowerDatacenter datacenter, java.util.List<Vm> vms, double lastClock, java.lang.String experimentName, boolean outputInCsv, java.lang.String outputFolder)` Prints the results. |
    | `static void` | `writeDataColumn(java.util.List<? extends java.lang.Number> data, java.lang.String outputPath)` Write data column. |
    | `static void` | `writeDataRow(java.lang.String data, java.lang.String outputPath)` Write data row. |
    | `static void` | `writeMetricHistory(java.util.List<? extends Host> hosts, PowerVmAllocationPolicyMigrationAbstract vmAllocationPolicy, java.lang.String outputPath)` Write metric history. |

    - ### Methods inherited from class java.lang.Object

      `clone, equals, finalize, getClass, hashCode, notify, notifyAll, toString, wait, wait, wait`

- - ### Constructor Detail


    - #### Helper

      ```
      public Helper()
      ```
  - ### Method Detail


    - #### createVmList

      ```
      public static java.util.List<Vm> createVmList(int brokerId,
                                    int vmsNumber)
      ```

      Creates the vm list.

      Parameters:
      :   `brokerId` - the broker id
      :   `vmsNumber` - the vms number

      Returns:
      :   the list< vm>


    - #### createHostList

      ```
      public static java.util.List<PowerHost> createHostList(int hostsNumber)
      ```

      Creates the host list.

      Parameters:
      :   `hostsNumber` - the hosts number

      Returns:
      :   the list< power host>


    - #### createBroker

      ```
      public static DatacenterBroker createBroker()
      ```

      Creates the broker.

      Returns:
      :   the datacenter broker


    - #### createDatacenter

      ```
      public static Datacenter createDatacenter(java.lang.String name,
                                java.lang.Class<? extends Datacenter> datacenterClass,
                                java.util.List<PowerHost> hostList,
                                VmAllocationPolicy vmAllocationPolicy)
                                         throws java.lang.Exception
      ```

      Creates the datacenter.

      Parameters:
      :   `name` - the name
      :   `datacenterClass` - the datacenter class
      :   `hostList` - the host list
      :   `vmAllocationPolicy` - the vm allocation policy
      :   `simulationLength` -

      Returns:
      :   the power datacenter

      Throws:
      :   `java.lang.Exception` - the exception


    - #### getTimesBeforeHostShutdown

      ```
      public static java.util.List<java.lang.Double> getTimesBeforeHostShutdown(java.util.List<Host> hosts)
      ```

      Gets the times before host shutdown.

      Parameters:
      :   `hosts` - the hosts

      Returns:
      :   the times before host shutdown


    - #### getTimesBeforeVmMigration

      ```
      public static java.util.List<java.lang.Double> getTimesBeforeVmMigration(java.util.List<Vm> vms)
      ```

      Gets the times before vm migration.

      Parameters:
      :   `vms` - the vms

      Returns:
      :   the times before vm migration


    - #### printResults

      ```
      public static void printResults(PowerDatacenter datacenter,
                      java.util.List<Vm> vms,
                      double lastClock,
                      java.lang.String experimentName,
                      boolean outputInCsv,
                      java.lang.String outputFolder)
      ```

      Prints the results.

      Parameters:
      :   `datacenter` - the datacenter
      :   `lastClock` - the last clock
      :   `experimentName` - the experiment name
      :   `outputInCsv` - the output in csv
      :   `outputFolder` - the output folder


    - #### parseExperimentName

      ```
      public static java.lang.String parseExperimentName(java.lang.String name)
      ```

      Parses the experiment name.

      Parameters:
      :   `name` - the name

      Returns:
      :   the string


    - #### getSlaTimePerActiveHost

      ```
      protected static double getSlaTimePerActiveHost(java.util.List<Host> hosts)
      ```

      Gets the sla time per active host.

      Parameters:
      :   `hosts` - the hosts

      Returns:
      :   the sla time per active host


    - #### getSlaTimePerHost

      ```
      protected static double getSlaTimePerHost(java.util.List<Host> hosts)
      ```

      Gets the sla time per host.

      Parameters:
      :   `hosts` - the hosts

      Returns:
      :   the sla time per host


    - #### getSlaMetrics

      ```
      protected static java.util.Map<java.lang.String,java.lang.Double> getSlaMetrics(java.util.List<Vm> vms)
      ```

      Gets the sla metrics.

      Parameters:
      :   `vms` - the vms

      Returns:
      :   the sla metrics


    - #### writeDataColumn

      ```
      public static void writeDataColumn(java.util.List<? extends java.lang.Number> data,
                         java.lang.String outputPath)
      ```

      Write data column.

      Parameters:
      :   `data` - the data
      :   `outputPath` - the output path


    - #### writeDataRow

      ```
      public static void writeDataRow(java.lang.String data,
                      java.lang.String outputPath)
      ```

      Write data row.

      Parameters:
      :   `data` - the data
      :   `outputPath` - the output path


    - #### writeMetricHistory

      ```
      public static void writeMetricHistory(java.util.List<? extends Host> hosts,
                            PowerVmAllocationPolicyMigrationAbstract vmAllocationPolicy,
                            java.lang.String outputPath)
      ```

      Write metric history.

      Parameters:
      :   `hosts` - the hosts
      :   `vmAllocationPolicy` - the vm allocation policy
      :   `outputPath` - the output path


    - #### printCloudletList

      ```
      public static void printCloudletList(java.util.List<Cloudlet> list)
      ```

      Prints the Cloudlet objects.

      Parameters:
      :   `list` - list of Cloudlets


    - #### printMetricHistory

      ```
      public static void printMetricHistory(java.util.List<? extends Host> hosts,
                            PowerVmAllocationPolicyMigrationAbstract vmAllocationPolicy)
      ```

      Prints the metric history.

      Parameters:
      :   `hosts` - the hosts
      :   `vmAllocationPolicy` - the vm allocation policy


- Overview
- Package
- Class
- Use
- Tree
- Deprecated
- Index
- Help

- Prev Class
- Next Class

- Frames
- No Frames

- All Classes

- Summary:
- Nested |
- Field |
- Constr |
- Method

- Detail:
- Field |
- Constr |
- Method
